# Capsaicin mediates caspases activation and induces apoptosis through P38 and JNK MAPK pathways in human renal carcinoma

**DOI:** 10.1186/s12885-016-2831-y

**Published:** 2016-10-12

**Authors:** Tao Liu, Gang Wang, Huangheng Tao, Zhonghua Yang, Yongzhi Wang, Zhe Meng, Rui Cao, Yu Xiao, Xinghuan Wang, Jiajie Zhou

**Affiliations:** 1 Department of Urology, Jingzhou Central Hospital, the Second Clinical Medical College, Yangtze University, Jingzhou, 434020 China; 2 Department of Urology, Zhongnan Hospital of Wuhan University, Center for Medical Science Research, Zhongnan Hospital of Wuhan University, Wuhan, 430071 China; 3 Department of Endodontics, Stomatology of Wuhan University, Wuhan, 430079 China

**Keywords:** Capsaicin, Renal cell carcinoma, TRPV1, Caspases, Apoptosis, MAPK

## Abstract

**Background:**

Renal cell carcinoma (RCC) is one of the tumors most refractory to chemotherapy to date. Therefore, novel therapeutic agents are urgently needed for this disease. Capsaicin (CPS), a natural active ingredient of green and red peppers, and a ligand of transient receptor potential vanilloid type 1 (TRPV1), has been showed potential in suppression of tumorigenesis of several cancers. Nonetheless, the anti-cancer activity of CPS has never been studied in human RCC.

**Methods:**

CCK8 analysis, LDH release activity and ROS generation analysis, flow cytometry analysis, and nuclear staining test were performed to test the influence of CPS in cultured cells in vitro, meanwhile western blot was done to uncover the precise molecular mechanisms. 786-O renal cancer xenografts were builded to investigate the antitumor activity of CPS in vivo.

**Results:**

We found treatment of CPS reduced proliferation of renal carcinoma cells, which could be attenuated by TRPV1 representative antagonist capsazepine (CPZ). CPS induced obvious apoptosis in renal carcinoma cells. These events were associated with substantial up-regulation of pro-apoptotic genes including c-myc, FADD, Bax and cleaved-caspase-3, -8, and -9, while down-regulation of anti-apoptotic gene Bcl2. Besides, CPS-treatment activated P38 and JNK MAPK pathways, yet P38 and JNK inhibitors afforded protection against CPS-induced apoptosis by abolishing activation of caspase-3, -8, and -9. Furthermore, CPS significantly slowed the growth of 786-O renal cancer xenografts in vivo.

**Conclusions:**

Such results reveal that CPS is an efficient and potential drug for management of human RCC.

**Electronic supplementary material:**

The online version of this article (doi:10.1186/s12885-016-2831-y) contains supplementary material, which is available to authorized users.

## Background

As the most common type of kidney cancer, renal cell carcinoma (RCC) is a worldwide public concern. Human RCC accounts for 2 to 3 % of all cancers, and approximately 90 % of kidney cancer, with an increasing incidence of diagnosis (representing 200,000 patients diagnosed per year worldwide) [1]. Unluckily, one third of the patients with RCC are diagnosed with metastatic or advanced diseases in a late stage, with a horribly high mortality rate counting approximately 100,000 deaths annually [1]. In the last decade, immunotherapy has become the major therapeutic option, yet with a low response rate (less than 20 %) [2]. Recently, molecular targeted small-molecule anti-cancer drugs such as sunitinib and sorafenib have shown a much better response in mRCC, however this therapy has been found to be associated with the development of renal impairment and the overall survival is still not satisfied [3]. Thus, novel anti-cancer agents for RCC (especially for mRCC) with high efficiency and safety are urgently needed.

Ion channels have a controversial role in cell proliferation and numerous studies have strongly confirmed that ion channels participated in nearly all basic cellular behaviors, including proliferation, differentiation, and apoptosis [4]. Transient receptor potential vanilloid type 1 (TRPV1) is a nonselective, ligand-gated cationic channel which can be regulated by thermal, mechanical and chemical stimuli such as CPS [5]. Primarily found in small-diameter nociceptive neurons, TRPV1 was subsequently found to be expressed in non-neuronal tissues such as bladder, cornea and renal [6–8]. Besides, TRPV1 was found to play important physiologic and pathological roles in renal, and its activation would make much improvement in acute and chronic renal diseases [9, 10].

Phytochemicals, particularly those included in the human diet, are ideal candidates for antitumorigenesis, antimutagenesis, or chemotherapy. CPS is a major pungent ingredient in red and green peppers which are widely used as spice [11]. CPS can activate TRPV1 channel specifically, inducing a temperary transient Ca^2+^ flux and an increase in cytosolic calcium concentration [12]. Emerging evidence shows that CPS has the potential to selectively suppress the growth of various human tumor cells in culture, including gastric [13] and prostate cancer cells [14]. What’s more, CPS has also exhibited anti-cancer effects in animal models, suppressing carcinogenesis of the colon [15], and lung [16]. However, the effects of CPS on renal cancer have never been investigated before. In this paper, the human renal carcinoma cell lines were selected to investigate whether CPS has anti-cancer effects on RCC and further clarify its mechanisms.

## Methods

### Cell culture

The human renal cell carcinoma 786-O, ACHN, Caki-1 cells (American Type Culture Collection, Manassas, VA) were cultured in RPMI 1640 medium containing 10 % fetal bovine serum (FBS, Gibco), 100 U/ml penicillin-G sodium and 100 μg/ml streptomycin sulfate at 37 °C under an atmosphere of 5 % CO2.

### Reagents and antibodies

CPS ([N-(4-hydroxy-3-methoxy-phenyl)methyl]-8-methyl-6-nonenamide), and capsazepine (CPZ) (N-[2-(4-chlorophenyl)ethyl]-1,3,4,5-tetrahydro-7,8-dihydroxy-2H -2-benzazepine-2-carbothioamide), were purchased from Cayman Chemical Company (Michigan, USA). CCK-8 (Roche Biochemicals, Mannheim, Germany) , and dimethyl sulfoxide (DMSO) were purchased from Sigma-Aldrich (St. Louis, MO). Anti-TRPV1 specific antibody (1:200, #ACC-030) was purchased from Alomone Labs (Jerusalem, Israel) and anti-GAPDH specific antibody (1:1000, sc-166574) was purchased from Santa Cruz Biotechnology (USA). Antibody against c-myc (1:1000, #ab32072) was from Abcam (Cambridge, UK). Antibodies against Bcl2 (1:1000, #12789-1-AP), Bax (1:1000, #50599-2-Ig) were purchased from Proteintech (Chicago, IL, USA). Antibodies against p-ERK1/2 (1:2000, #4370P), total ERK1/2 (1:2000, #4695P), p-JNK (1:1000, #4668P), total JNK (1:1000, #9258P), p-P38 (1:1000, #4511P), total P38 (1:1000, #9212P), FADD (1:1000, #2782), and caspase-3 (1:500, #9665), caspase-8 (1:1000, #9496), caspase-9 (1:1000, #9502) were obtained from Cell Signaling Technologies (Danvers, MA).

### RT-PCR

Total RNA was isolated from 786-O cells using the TRIzol Reagent (Invitrogen). For reverse transcriptase (RT) analysis, 3 μg of RNA was reverse transcribed into cDNA using RevertAid Fist Strand cDNA Synthesis Kit (Thermo Scientific). PCR was performed by adding 2 μl RT reaction mixture in a final volume of 50 μl. DNA amplification conditions included an initial 5 min denaturation step at 95 °C and 35 cycles of 30 s at 95 °C, 30 s at 60 °C, and 60 s at 72 °C, and finally 5 min at 72 °C. PCR primers used were designed as follows: TRPV1 forward primer: 5′-TTCCGA GGG ATT CAG TAT TT-3′ and reverse primer: 5′-TGA GCA GGA GGA TGT AGG TG-3′; β actin forward primer: 5′-AGA AGG ATT CCT ATG TGG GCG-3′and reverse primer: 5′-CAT GTC GTC CCA GTT GGT GAC-3′.

### Western blot assay

Cells were washed three times with ice-cold PBS and underwent lysis in 1 % Triton lysis buffer on ice, then quantified with BCA kit. For each sample, 10–50 g protein was separated on 10–15 % SDS-PAGE (Promoton Biotechnology, Shanghai, China) and transferred onto PVDF membrane (Millipore, Bedford). Membranes were incubated with primary antibodies overnight at 4 °C followed by HRP-conjugated secondary antibodies at 37 °C for 2 h, and developed with the WesternBright ECL HRP substrate (Advansta).

### CCK8 assay

Cell growth and viability were measured using cell proliferation and cytotoxicity reagent WST-8 (Roche Biochemicals, Mannheim, Germany). Briefly, the protocol was as follows: 786-O, ACHN, Caki-1 cells (5 × 10^3^ per well) were cultured in a 96-well plate. After 12 h (time for cells to attach to the plate surface), cells were treated with different concentrations of CPS (0–400 μM) or pretreated with CPZ (2 μM) for 2 h and then treated with CPS, ten wells each group for statistics. At the harvest time 10 μl of CCK8 was added into each well and after one hour’s incubation cell viability was determined by measuring the absorbance of the converted dye at 490 nm. The experiments were triplicate.

### Analysis of LDH release activity and ROS generation

Lactate dehydrogenase (LDH) release assay was employed to assess cell death according to the manufacturers’ recommendations (Beyotime Institute of Biotechnology, Nantong, China). The assay was quantitated by determining the absorbance at 490 nm. Reactive Oxygen Species (ROS)-sensitive fluorescent probe, DCFH-DA was used to assess intracellular production of ROS in 786-O, ACHN, and Caki-1 cells treated with CPS, following a microplate assay procedure. The non-fluorescent DCFH-DA probe enters a cell, and is hydrolyzed by cellular esterases, subsequently oxidated by ROS in the cell, which change to a fluorescent form. 786-O, ACHN, and Caki-1 cells were cultured in 96-well plate (1 × 10^4^ cells per well) and treated with different concentrations of CPS for 24 h, with/without being pretreated with CPZ. Afterwards, the probe DCFH-DA was added to a final concentration of 10 μM in RMPI 1640 medium and then incubated at 37 °C without exposure to light for 20 min. After washed at least three times with culture medium, fluorescence enzyme-linked immunoassay reader was used to measure the mean RFU of each plate (emission at 525 nm with excitation at 488 nm).

### Analysis of apoptosis with flow cytometry and hochest 33258 Nuclear Staining

For analysis of apoptosis, after indicated time of CPS incubation, 786-O, ACHN, and Caki-1 cells were collected with trypsin and incubated in a binding buffer containing FITC-conjugated annexin V and PI at room temperature for 5 min in the dark and detected with flow cytometry analysis. To visualize apoptotic bodies, 786-O cells were exposed to different concentrations of CPS for 24 h, fixed in 4 % paraformaldehyde and stained with 1 ml 10 μg/ml Hochest 33258 (Sigma) for 30 min at 37 °C in the dark. After thoroughly washed with PBS, the cells were checked for karyopyknosis under the inverted fluorescence microscope.

### In vivo animal treatment protocol

Athymic nude (nu/nu) 5-week-old mice were supplied by Experimental Centre of Medical Scientific Academy of Hubei province. The animal study was performed in the Animal Biosafety Level 3 Laboratory of Wuhan University (Wuhan, China) accredited by the AAALAC International. The animal protocol used in this study was approved by local ethics committee (Ethical Committee of Zhongnan Hospital, Wuhan University, IRB ID: 2012030), and was in accordance with the ARRIVE guidelines and the Guide for the Care and Use of Laboratory Animals (eighth edition) by the National Research Council of the United States National Academies. All mice were housed in alaminar air-flow cabinet under pathogen-free conditions with a 12 h light/dark schedule at controlled temperature and humidity with food and water ad libitum. After acclimated for one week prior to study initiation, mice were then were injected subcutaneously into the right flank with 1 × 10^7^ 786-O cells in 0.1 ml of sterile PBS. Six weeks post-inoculation, mice were divided into two experimental groups (*n* =10 per group) based on the initial tumor volume, the CPS group with treatment of CPS 5 mg/kg in 100 μl of PBS containing 0.1 % DMSO and the control group received 100ul PBS containing 0.1 % DMSO. The injection was repeated by gavage every three days for a total of 4 weeks until the mice were sacrificed. Mice were monitored daily for tumor growth (using digital calipers), hair coat, overall activity and body weight. Then, all the mice were sacrificed by cervical dislocation, under anaesthetization using diethyl ether through inhalation. Tumor volumes were calculated by the formula: *1/2(Length × Width* ^*2*^ *)*. The proliferating tumor cells were detected by proliferating cell nuclear antigen (PCNA) and Ki-67 staining. The evaluation of PCNA and Ki-67 expression was based on the proportion of positively stained cells, in which the cell nuclei were stained dark brown, among a total of 1000 counted cells. The apoptosis rate was determined by terminal deoxynucleotidyl transferase (TdT) dUTP nick-end labelling (TUNEL) reaction technique, and the percentage of labeled nuclei was then calculated and defined as numbers of brown apoptosis bodies in each field. All sections of this report adhere to the ARRIVE Guidelines for reporting animal research [17]. A completed ARRIVE guidelines checklist is included in Additional file 1: Checklist S1.

### Statistical analysis

SPSS version 13.0 (University of Nevada, Las Vegas, NV, USA) was used for the statistical analysis. All data is presented as mean ± SD. Statistical analysis was performed using One-Way ANOVA, with *P* < 0.05 taken as statistically significant.

## Results

### CPS decreased the viability of 786-O cells

**Fig. 1:**
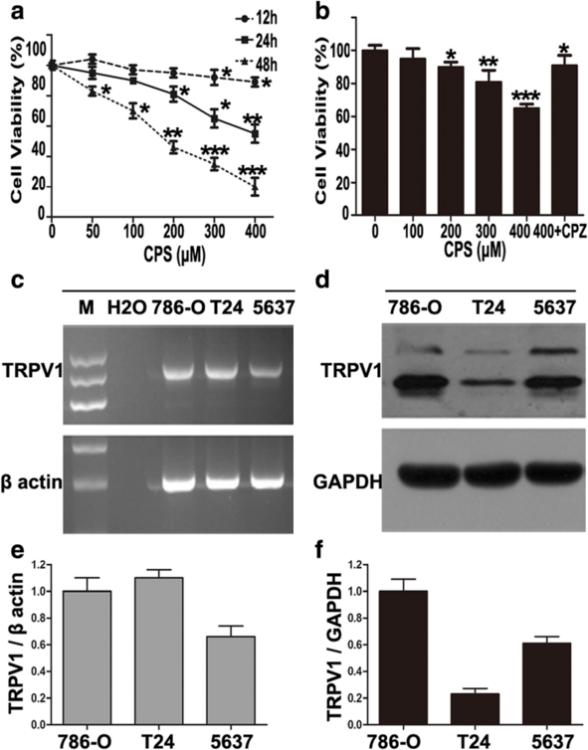
CPS decreased the viability of 786-O cells. **a** Cell viabilities were determined after 786-O cells was incubated with vehicle (0.1 % DMSO) or different concentrations of CPS for 12, 24, 48 h by CCK8 assay, and are expressed as percent against control, which was taken as 100 %, and treated with medium-containing vehicle (0.1 % DMSO). **b** Pretreatment of CPZ at 2 μM for 2 h significantly attenuated the decreasing of cell viability by 48 h incubation of CPS. All of the CCK8 assays were conducted in triplicate. **c** and **d** The expression of TRPV1 in 786-O, T24, and 5637 cells was detected by RT-PCR (**c**) and western blot (**d**). β actin and GAPDH were used as internal standards in RT-PCR and western blot, respectively. **e** and **f** TRPV1 expression was quantified and the results of RT-PCR (**e**) and western blot (**f**) were presented in histograms. Expression of TRPV1 in different cells was normalized over 786-O cells. *P < 0.05, **P < 0.01, ***P < 0.001; bars, SD. One-Way ANOVA was used for the data analysis

The cytotoxic effect on cell proliferation of CPS on 786-O cells was measured by CCK8 assays. 786-O cells were treated with varying concentrations (from 0 to 400 μM) of CPS for 12, 24 or 48 h, respectively. Cell viability curves showed that CPS inhibited the proliferation of 786-O cells in both dose- and time-dependent manners. In 48 h group, cell viability in DMSO-treated cells was taken as 100 %, and CPS at 50, 100, 200, 300 and 400 μM decreased the cell viability by 7.20, 15.91, 50.96, 62.40, and 76.77 %, respectively, which indicated an approximate IC_50_ of 200 μM. And when cells were incubated with 200 μM CPS, cell viabilities at 12, 24, and 48 h were decreased by 4.2, 20.77, and 50.90 %, respectively (Fig. 1a). The proliferation of ACHN, and Caki-1 cells were also found to be inhibited by CPS in a dose-dependent manner (Additional file 2: Figure S1).

We then detected whether the anti-proliferative effect of CPS in our model also referred to its targeted binding to TRPV1. As shown in Fig. 1b, pretreated CPZ at 2 μM for 2 h partially attenuated the cytotoxic effect of CPS, indicating that TRPV1 partially mediated CPS-induced proliferative suppression. Besides, we also detected expression of TRPV1 in 786-O cells, finding that TRPV1 expressed in 786-O at both mRNA and protein levels (Fig. 1c, d, e and f).

### CPS increased LDH release activity and ROS generation

**Fig. 2:**
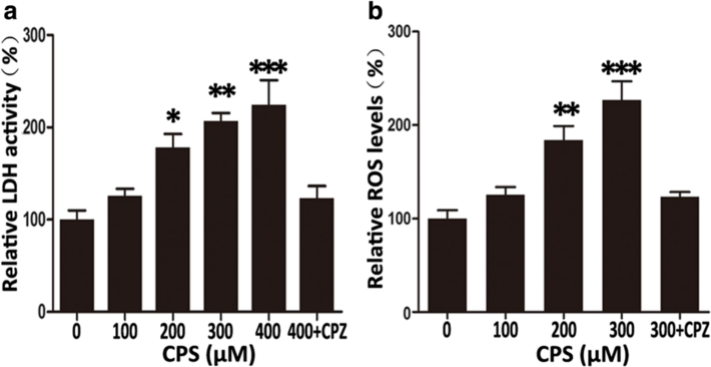
CPS increased LDH release activity and ROS Generation in 786-O cells. **a** LDH release assays with 786-O cells treated with different concentrations of CPS (from 100 to 400 μM) for 72 h, or with 400 μM CPS for 72 h after being pretreated with 2 μM CPZ for 2 h. **b** DCFH-DA fluorescence images are shown with the fluorescence intensity representing ROS concentration. Cells stimulated with vary concentrations (from 100 to 300 μM) of CPS for 24 h, or with 300 μM CPS after being pretreated with CPZ. Statistical data from multiple experiments (mean ± SD, *n* = 30). *P < 0.05, ** *P* < 0.01, ***P < 0.001; bars, SD. One-Way ANOVA was used for the data analysis

The cytotoxic activity of CPS was further confirmed by LDH release assays. Increased LDH activity was observed in CPS-treated 786-O cells in a dose-dependent manner (Fig. 2a). Compared with normal control, 200, 300, and 400 μM of CPS increased the LDH release activity of 786-O cells by 77.58, 106.87, and 124.33 %, respectively, whereas there were no significant changes in cells treated with CPS + CPZ (pretreatment of 2 μM CPZ for 2 h, and then 400 μM CPS for another 72 h) or cells treated with DMSO or 100 μM CPS (Fig. 2a). We also determined whether CPS induced generation of ROS both by ROS Kit and flow cytometry analysis. In ROS Kit assays, after stimulation, CPS at 100, 200, and 300 μM increased the ROS generation by 25.35, 83.74, and 162.80 %, respectively, which could be partially abolished by CPZ (Fig. 2b). By flow cytometry analysis, we found CPS induced significant increase of ROS generation in 786-O cells at the concentration of 200, and 300 μM, and in ACHN cells of 300 μM, while in Caki-1 cells CPS failed to induce significant increase of ROS generation (Additional file 3: Figure S2).

### CPS induced apoptosis in 786-O cells

**Fig. 3:**
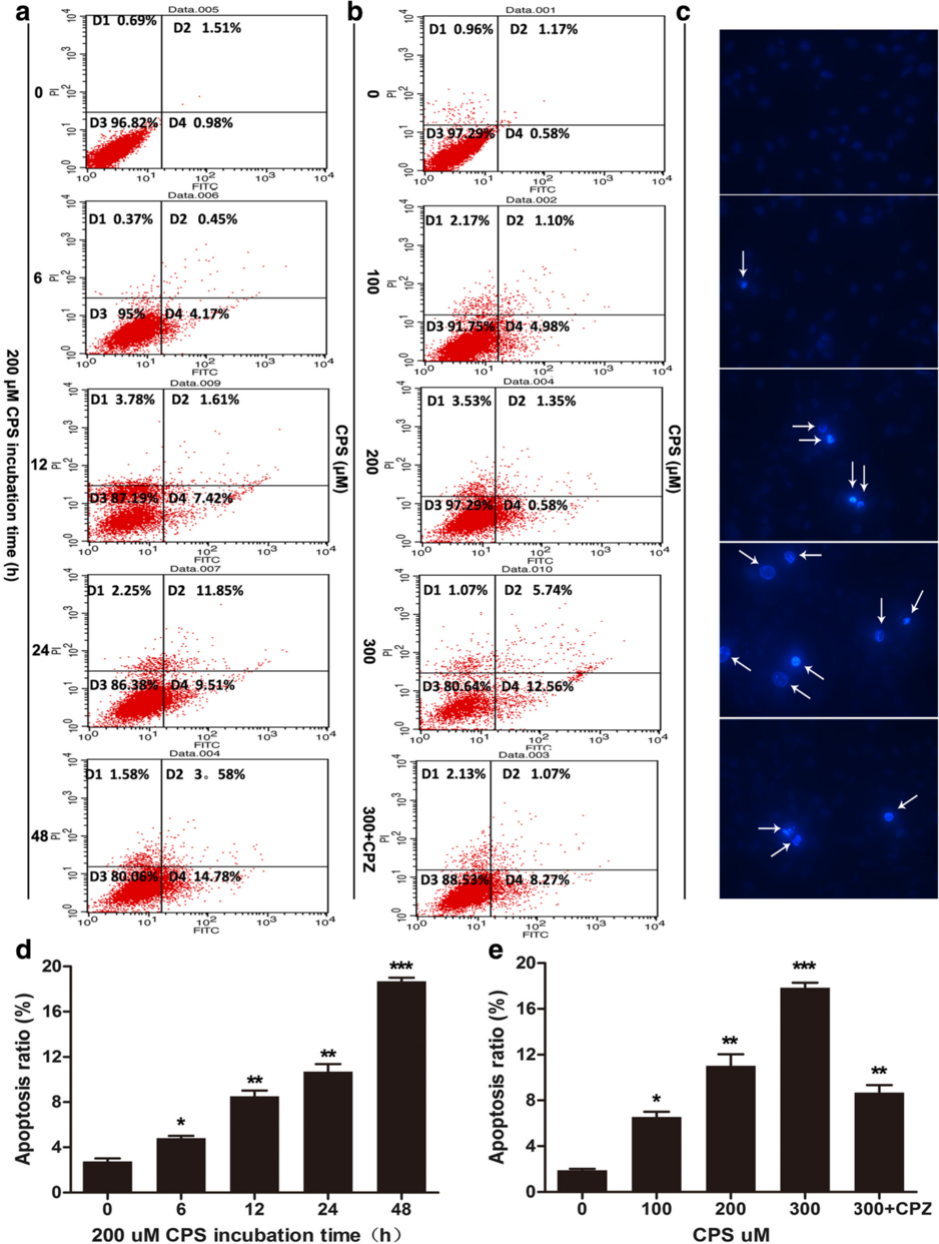
CPS induced apoptosis in 786-O cells. **a** and **b** 786-O cells were treated with vary concentrations of CPS, or pretreated with 2 μM CPZ for 2 h and then treated with 300 μM CPS for 24 h (**b**), or treated with 300 μM CPS for 0, 6, 12, 24, or 48 h (**a**). Then, cells were collected and incubated in a binding buffer containing FITC-conjugated annexin-V, and PI was then detected with flow cytometry analysis. **c** The hochest 33258 staining assay revealed that CPS facilitated cell apoptosis in 786-O cells. Arrows indicate apoptotic cells. **d** and **e** The quantitative data showed the percentage of apoptotic cells in A and B. *P < 0.05, ** *P* < 0.01, ***P < 0.001; bars, SD. One-Way ANOVA was used for the data analysis

In order to ascertain the precise mechanisms of the anti-cancer activity of CPS, cell apoptosis was detected by flow cytometry analysis. As shown in Fig. 3a and b, CPS triggered apoptosis in both time- and dose-dependent manners. At the concentration of 200 μM, in comparison with the control, CPS increased apoptosis by 1.13, 6.54, 8.88, and 15.87 % after 6, 12, 24, 48 h incubation, respectively (Fig. 3a and d). As shown in Fig. 3b and e, CPS also caused cell apoptosis in a dose-dependent manner, and at the dose of 0, 100, 200 and 300 μM, CPS induced 1.80, 6.10, 9.30 and 12.00 % apoptosis, respectively, after 24 h treatment. However, CPZ could also decrease CPS-induced apoptosis (Fig. 3e). To further confirm apoptotic cell death, we examined nuclear morphological changes using Hochest 33258 staining. After stimulation of CPS, 786-O cells exhibited obviously condensed and fragmented nuclei, while CPZ partially reverted the change of cell morphology (Fig. 3c). In addition, CPS decreased the expression of Ki-67 but increased the TUNEL staining in RCC cells (Additional file 4: Figure S3). These data clearly demonstrated that CPS induced apoptosis in 786-O cells.

**Fig. 4:**
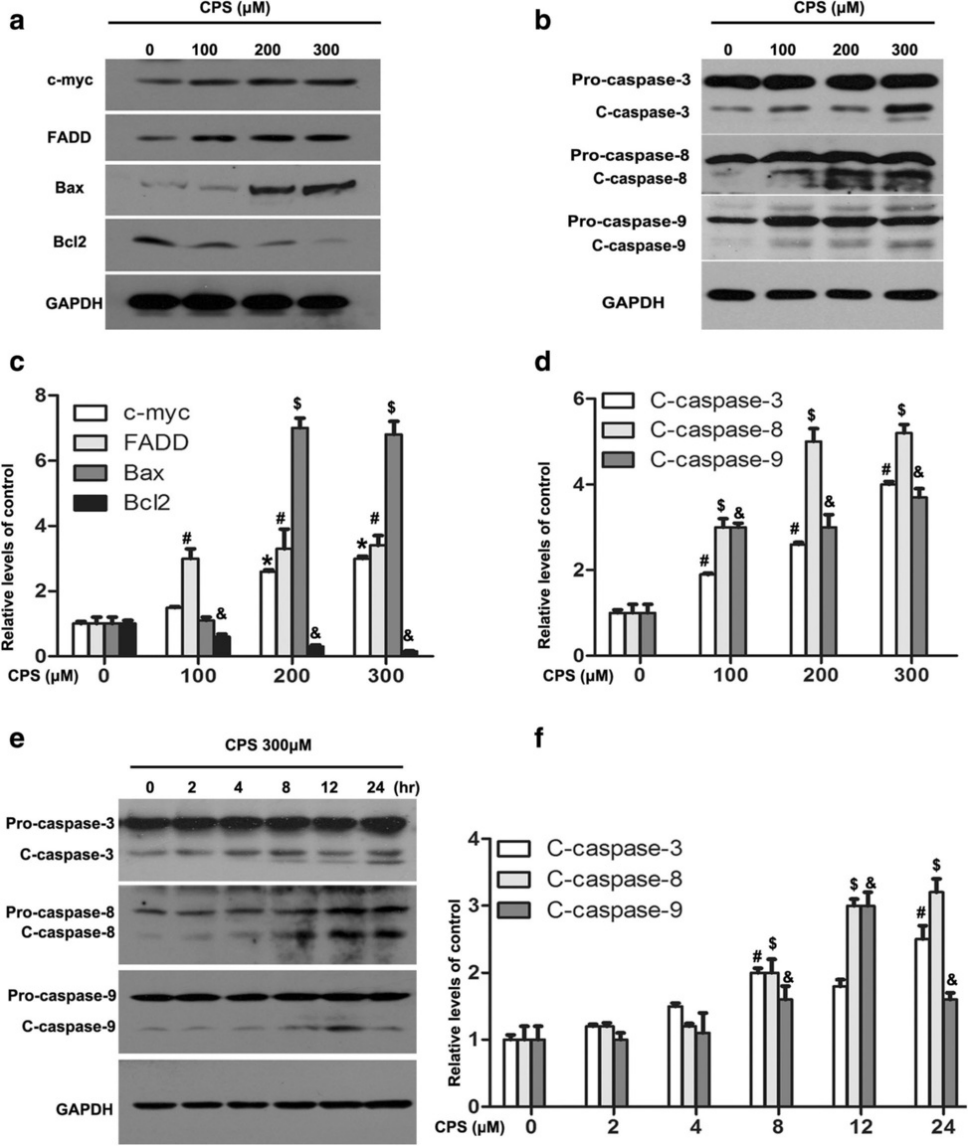
Alterations of apoptosis related proteins in 786-O cells after treated with CPS. **a** and **b** After 48 h CPS treatment with vary concentrations of CPS, c-myc, FADD, Bax, Bcl2, Caspase-3, -8, and -9 were analyzed in each sample by western blot. **c** Caspase-3, -8, and -9 were analyzed by western blot after incubation of 300 μM CPS for 0, 2, 4, 8, 12, 24 h. **d**, **e**, and **f** The quantitative data of the western blot of (**a**), (**b**), and (**c**), respectively. The values of each indicated protein mean relative density of the band normalized to GAPDH. Values represent the mean ± SD of three independent experiments. *^,#,$,^&*P* < 0.05 compared to the vehicle control groups, and statistic analysis was performed by One-Way ANOVA

In an attempt to gain further insight into the mechanism underlying CPS-induced apoptosis of 786-O cells, we investigated the protein levels of several key apoptosis-linked gene products, such as c-myc, FADD, Bcl2 and Bax. Expression of the pro-apoptotic c-myc, FADD, and Bax were up-regulated by CPS in a dose-dependent manner, while the anti-apoptotic Bcl2 down-regulated (Fig. 4a and c). Caspase families including caspase-3, -8, and -9 were also tested by western blot assays. As Fig. 4b and d showed, exposure to CPS (0–300 μM) for 24 h caused dose-dependent increase of the cleaved fragments of caspase-3, -8, and -9. Moreover, CPS (200 μM) treatment also resulted in a time-dependent increase of cleaved caspase-3, -8, and -9 (Fig. 4e and f). These results suggested that CPS could modulate the activation of the caspase apoptotic signaling pathways in 786-O cells.

### P38 and JNK MAPK signaling pathways mediated CPS-induced apoptosis

**Fig. 5:**
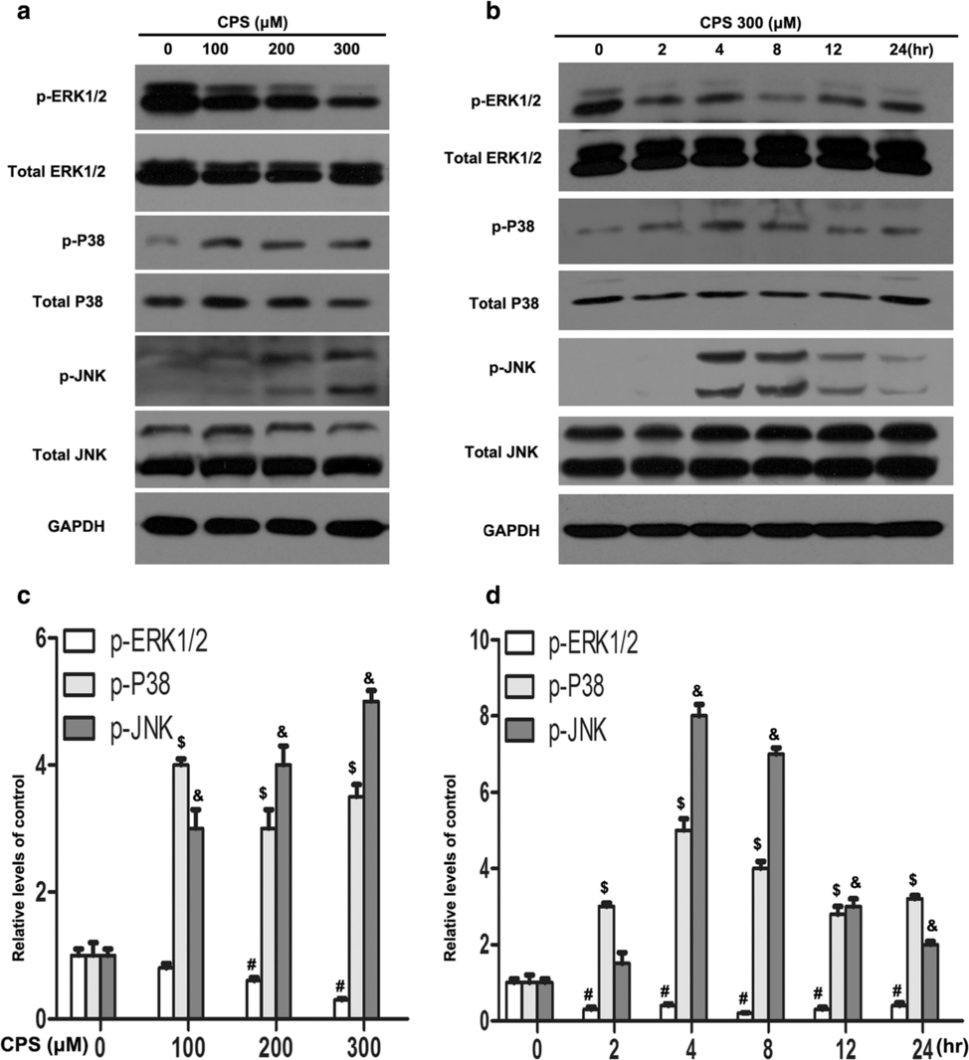
The changes of MAPK signal pathways. **a** After 48 h CPS treatment with vary concentrations of CPS, p-ERK1/2, total ERK1/2, p-P38, total P38, p-JNK, and total JNK were analyzed by western blot. **b** Indicated proteins were analyzed after incubation of 300 μM CPS for 0, 2, 4, 8, 12, 24 h. (**c**) and (**d**) The quantitative data of the western blot of **a**, and **b**, respectively. The values of each indicated protein mean relative density of the band normalized to GAPDH. The Figures are representative of three experiments. Values represent the mean ± SD of three independent experiments. The data are presented as mean ± SD. ^#,$,^&*P* < 0.05, bars, SD. One-Way ANOVA was used for the data analysis

**Fig. 6:**
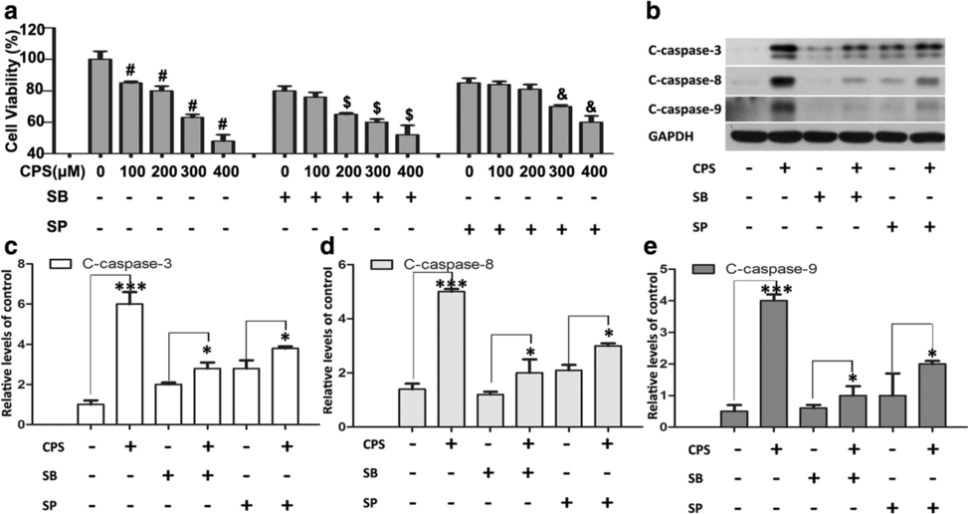
P38 and JNK inhibitors afforded protection against CPS-induced apoptosis. **a** 786-O cells were treated by monotherapy of CPS, or combined therapy of CPS with pretreatment of P38 inhibitor (SB203580, 20 μM) or JNK inhibitor (SP600125, 10 μM) for 4 h, respectively, Then, cell viability was measured by CCK8 assays. **b** Pretreatment of SB203580 or SP600125 inhibited the caspases activation induced by CPS, which was measured by western blot. **c**, **d** and **e** The results of the western blot were quantified and expressed in histograms. The results were quantitated by densitometric analysis. The Figures are representative of three experiments. The data are presented as mean ± SD. The quantitative data of the western blot and statistic analysis was performed by One-Way ANOVA. *P < 0.05, **P < 0.01, ***P < 0.001; bars, SD

Further, we determined whether MAPKs were implicated in the anti-cancer effect of CPS on 786-O cells. We found that p-ERK1/2 (phosphorylated form of ERK1/2) was decreased, while p-P38 and p-JNK (phosphorylated form of P38 and JNK) increased substantially in cells treated with CPS, in a dose-dependent manner (Fig. 5a and c). Moreover, CPS (200uM) treatment also presented a time-dependent decrease of p-ERK1/2, yet a time-dependent increase of p-P38 and p-JNK (Fig. 5b and d). To confirm the involvement of the MAPKs, we analyzed the effects of the P38 inhibitor (SB203580, 20 μM) and JNK inhibitor (SP600125, 10 μM) on the CPS-mediated proliferative inhibition by CCK8 assays. The anti-proliferative activity was dramatically affected by SB203580 and SP600125 (Fig. 6a).

Next, we further investigated relationships between CPS-induced alteration of MAPKs and activation of caspase-3, -8, and -9. 786-O cells were pretreated with SB203580, or SP600125 for 4 h, treated with 300 μM CPS for another 24 h, and then analyzed by western blot. As shown in Fig. 6b, c, d and e, treatment of SB203580, or SP600125 significantly attenuated CPS-induced activation of caspase-3, -8, and -9, which suggested that activation of P38 and JNK MAPKs might play a critical upstream role in mediating CPS-induced caspases activation.

### CPS slowed the growth of 786-O xenografts in vivo

**Fig. 7:**
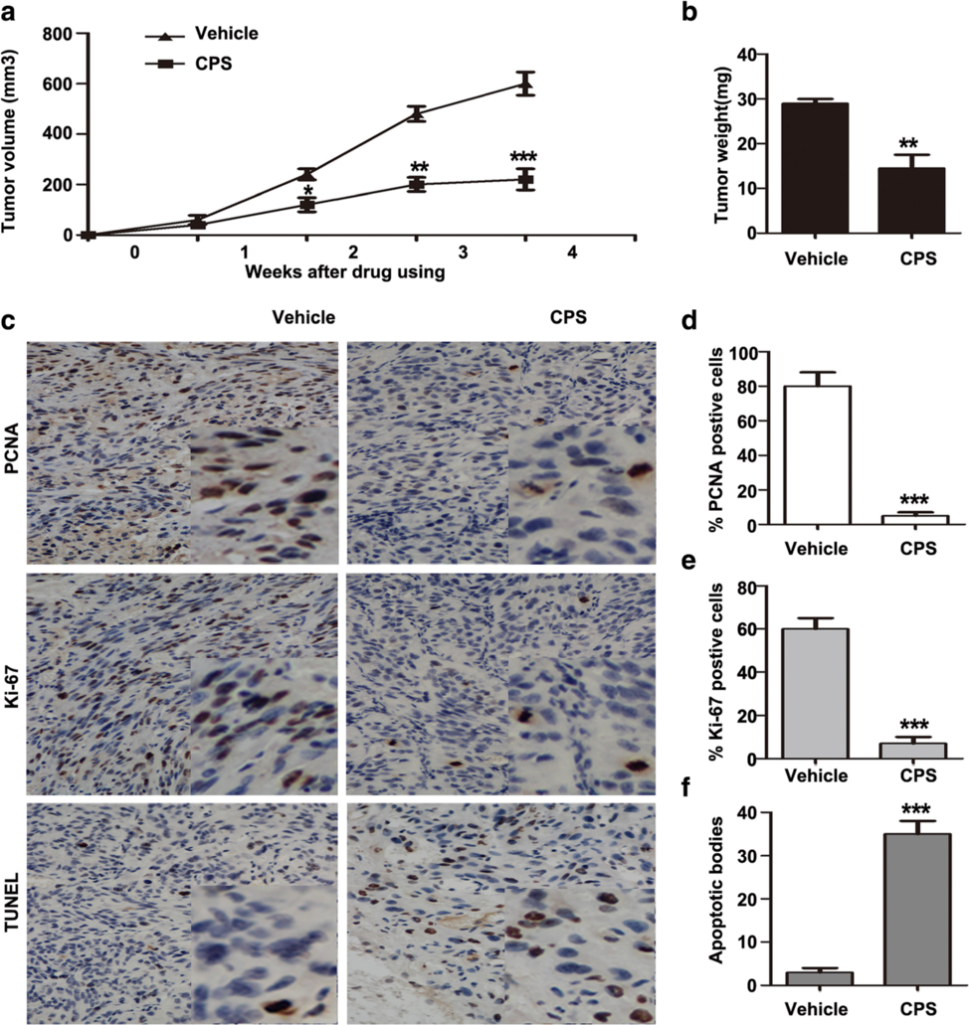
CPS slowed the growth of 786-O xenografts in vivo. **a** and **b** CPS suppressed the growth of 786-O xenografts in nude mice models by tumor volume (**a**) and tumor weight (**b**). **c** Tumor sections were immunostained for PCNA (*top panels*) and Ki-67 to assess cell proliferation (*medium panels*), and stained with TUNEL to assess the apoptosis rate (*bottom panels*). **d**, **e** and **f** Quantitation of PCNA , Ki-67 and TUNEL positive cells indicated that the administration of CPS reduced cell proliferation and increased apoptosis in 786-O tumors in vivo, relative to controls. The data are presented as mean ± SD. *P < 0.05, ** *P* < 0.01, ***P < 0.001; bars, SD. One-Way ANOVA was used for the data analysis

To extend the observations made in cultured cells and to assess the efficacy of CPS in vivo, its effect on the growth of 786-O renal tumor xenografts was determined in athymic nude mice. 786-O xenograft models showed dramatic response to CPS, with a significant reduction in tumor volume and weight (Fig. 7a and b), on comparison with vehicle treatment. The mean body weight and hair coats, as well as overall activity, were similar in both groups at the termination of the experiment, suggesting that CPS had no major negative effects on these mice (data not shown). As a result, tumors from CPS-treated mice exhibited notably lower expression of PCNA and Ki67 by immunohistochemical staining compared with tumors from control mice (Fig. 7c, d, and e), which confirmed a significant decrease in proliferation of tumor xenografts by CPS. In addition, higher count of brown apoptotic bodies were observed in tumors from CPS-treated mice by TUNEL, suggesting apoptosis was a possible mechanism for tumor growth inhibition (Fig. 7c, and f). These results positively corresponded with our in vitro studies.

## Discussion

RCC is one of the most refractory tumors to chemotherapy to date. Therefore, novel therapeutic agents are urgently needed for this disease. In the last decade, the role of CPS in tumoral growth and cell transformation represents a much-discussed question in scientific literature [18, 19]. CPS shows the ability to induce apoptosis and cell cycle arrest and suppress metabolic activation in transformed cells [20, 21]. However, whether CPS has anti-cancer activity in human RCC keeps unknown. The present manuscript fills this void of knowledge and explores the chemopreventive potential of CPS in RCC 786-O cells. To our knowledge, our pilot study showed for the first time that CPS displayed potent anti-cancer activity in human RCC. The anti-proliferative activity of CPS was confirmed by substantial increase of LDH release activity and ROS generation, which were partially attenuated by CPZ (a representative antagonist of TRPV1). As a nonselective and ligand-gated cationic channel receptor, TRPV1 had recently been shown involved in malignant cell growth and progression by controlling cell survival and apoptotic cell death [22, 23]. We found that TRPV1 expressed in 786-O at both mRNA and protein levels, and two bladder cancer lines T24 and 5637 were taken as the positive controls of TRPV1 expression, which were verified in the previous studies of our research team [24, 25]. These results suggested that CPS induced increase of LDH release activity and ROS generation partially via activating TRPV1.

Efforts have been made to develop chemoprevention strategies that trigger apoptosis of malignant cancer cells [26, 27]. Apoptosis, known as an encoded suicide program, is arguably the most potent natural defense against cancer [28], and resistance to apoptosis is considered as a hallmark of cancer. What’s more, the majority of studies exploring the anti-cancer activity of CPS have focused on the mechanisms underlying CPS-induced apoptosis [29, 30]. In the present study, we observed significant apoptosis of 786-O cells by CPS treatment, which was also attenuated by pre-treatment of CPZ. Besides, we found rapid up-regulation of pro-apoptotic genes including c-myc, FADD, Bax and, while down-regulation of anti-apoptotic gene Bcl2 which eventually led to the activation of caspase-3, -8, and -9 cascade, suggesting that CPS-induced apoptosis might partly occured through a mitochondrion-mediated pathway. Bcl2, an anti-apoptotic oncoprotein, has been shown to act on mitochondria and prevent the release of cytochrome c and thus caspases activation, while Bax mediated exactly opposite processes [31, 32]. The ratio of Bcl2 to Bax, rather than the levels of the individual proteins, is considered to be critical in determining the survival or death of cells [33]. We showed that Bcl2 was decreased while Bax increased thus the ratio of Bcl2 and Bax decreased dramatically by CPS, which may induce apoptotic response. In addition, apoptosis is also mediated by proteolytic enzymes called caspases, which trigger cell death by cleaving specific proteins in the cytoplasm and nucleus [34]. We observed that significant activation of caspase-3, -8 and -9 occurred after treatment with CPS.

Some evidence suggests that MAPK signaling pathways play an important role in the action of some chemotherapeutic drugs in the regulation of apoptosis [34, 35]. It was found that CPS could increase P38 phosphorylation, and JNK phosphorylation, thus promoting apoptosis [36, 37]. Some other papers also showed CPS could alter MAPK pathways [38]. On the basis of previous reports, we further investigated activation of MAPKs in our study. We found p-ERK1/2 down-regulated while p-P38 and p-JNK increased substantially in CPS-treated 786-O cells. Nonetheless, pre-treatment with P38 inhibitor (SB203580, 20uM) and JNK inhibitor (SP600125, 10 μM) effectively inhibited CPS-induced activation of caspase-3, -8, and -9, thus inhibited apoptosis and recovered cell viability. Taken together, these results suggested that activation of P38 and JNK MAPKs played an important role in CPS-induced apoptosis.

Whether these in vitro observations has any relevance to that in vivo was also investigated. Our results showed that CPS possessed a profound anti-proliferation effect on 786-O xenograft renal tumors without major side effects on these nude mice.

## Conclusions

In conclusion, we are the first to explore the anti-cancer potential of CPS in renal cell carcinoma. We found treatment of CPS reduced proliferation of 786-O cells, and induced obvious apoptosis. These events were associated with substantial up-regulation of pro-apoptotic genes including c-myc, FADD, Bax and cleaved-caspase-3, -8, and -9, while down-regulation of anti-apoptotic gene Bcl2. Besides, CPS-treatment activated P38 and JNK MAPK pathways, yet P38 and JNK inhibitors afforded protection against CPS-induced apoptosis by abolishing activation of caspase-3, -8, and -9. Furthermore, CPS significantly slowed the growth of 786-O renal cancer xenografts in vivo. Such results reveal that CPS is an efficient and potential drug for management of human RCC. Therefore, CPS shows brilliant antitumor properties, thus represents promising drugs against renal cell carcinoma and should thus be explored additionally for therapeutic use.

## Additional files

Additional file 1: NC3Rs ARRIVE Guidelines Checklist. (PDF 1527 kb)
Additional file 2: Figure S1. CPS decreased the viability and induced apoptosis of ACHN and Caki-1 cells. A: Cell viabilities were determined after ACHN and Caki-1 cells was incubated with vehicle (0.1 % DMSO) or different concentrations of CPS for 48 h by CCK8 assay, and are expressed as percent against control, which was taken as 100 %, and treated with medium-containing vehicle (0.1 % DMSO). B: ACHN and Caki-1 cells were treated with vary concentrations of CPS for 24 h. Then, cells were collected and analyzed by flow cytometry analysis. C: The quantitative data showed the percentage of apoptotic cells in (C). *^,#^ *P* < 0.05, **^,##,$$,^&&*P* < 0.01, ***^,###,$$$,^&&&*P* < 0.001; bars, SD. One-Way ANOVA was used for the data analysis. (JPG 800 kb)
Additional file 3: Figure S2. CPS increased ROS Generation in RCC cells. A: Flow cytometry analysis for 786-O, ACHN, and Caki-1 cells stained with DCFH-DA, and statistically analyzed in (B), revealing an increased ROS in the RCC cells. C: DCFH-DA stained (green) 786-O, ACHN, and Caki-1 cells after CPS and vehicle treatment. Nuclears were stained by DAPI (blue). The images were photographed by fluorescence microscope and scale bars for (C) are 20 μm. ^#^ *P* < 0.05, **P* < 0.01, ****P* < 0.001; bars, SD. One-Way ANOVA was used for the data analysis. (JPG 587 kb)
Additional file 4: Figure S3. CPS decreased the expression of Ki-67 but increased the TUNEL staining in RCC cells. A: Immunofluorescence staining of Ki-67 (green) in ACHN, and Caki-1 cells after CPS and vehicle treatment. B: Immunofluorescence staining of TUNEL (green) in 786-O, ACHN, and Caki-1 cells. Nuclears were stained by DAPI (blue). The images were photographed by fluorescence microscopy. The scale bar for C and D is 20 μm. (JPG 693 kb)

## Abbreviations

CPS: Capsaicin
CPZ: Capsazepine
DMSO: Dimethyl sulfoxide
RCC: Renal cell carcinoma
RT: Reverse transcriptase
TRPV1: Transient receptor potential vanilloid type 1
TUNEL: Terminal deoxynucleotidyl transferase (TdT) dUTP nick-end labelling
