# Supplementary figures and images for: Capsaicin mediates caspases activation and induces apoptosis through P38 and JNK MAPK pathways in human renal carcinoma
Source: BMC Cancer. 2016 Oct 12;16:790. doi: 10.1186/s12885-016-2831-y (PMC5059898; doi:10.1186/s12885-016-2831-y)

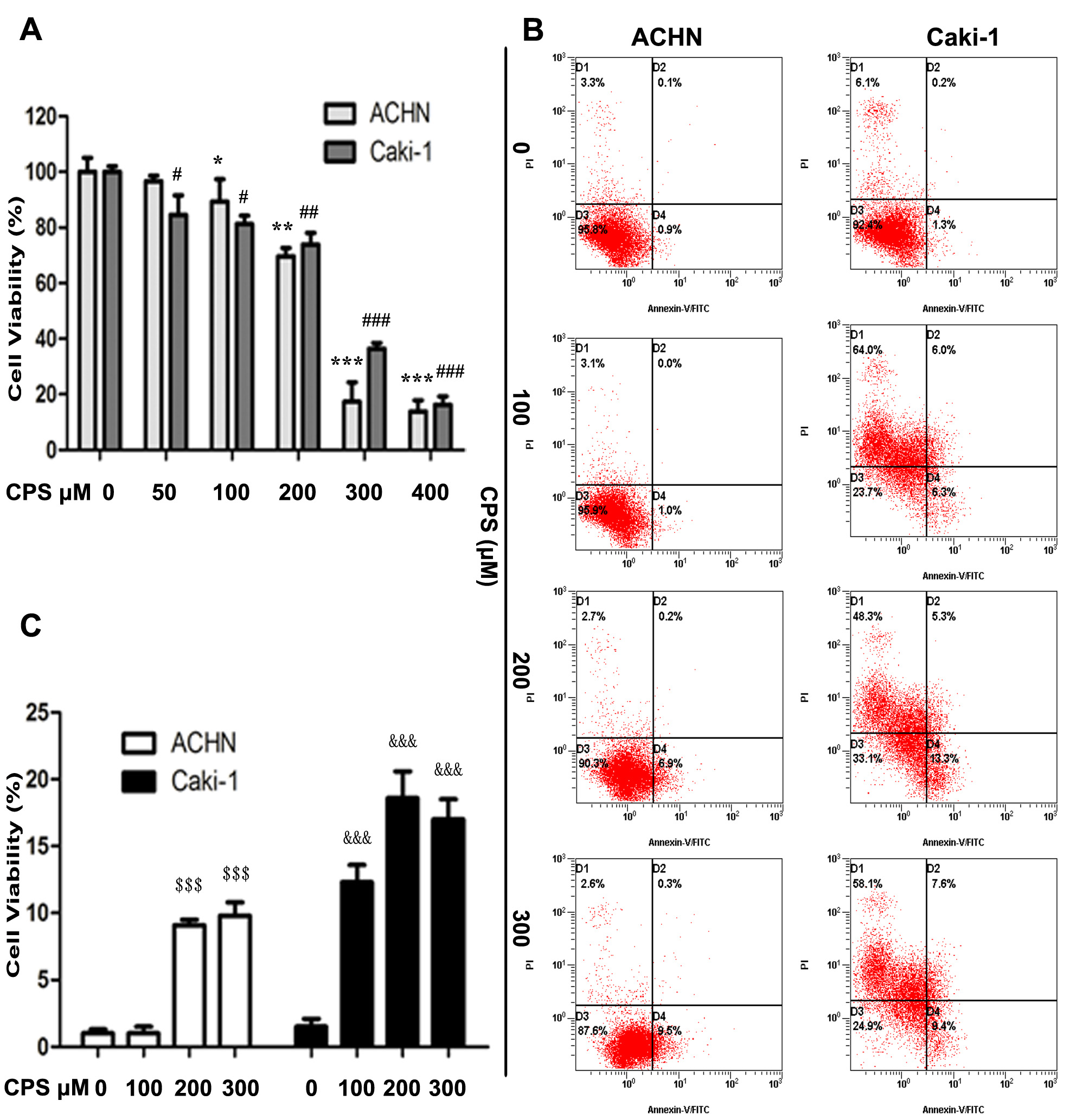

Supplement: Additional file 2: Figure S1. — CPS decreased the viability and induced apoptosis of ACHN and Caki-1 cells. A: Cell viabilities were determined after ACHN and Caki-1 cells was incubated with vehicle (0.1 % DMSO) or different concentrations of CPS for 48 h by CCK8 assay, and are expressed as percent against control, which was taken as 100 %, and treated with medium-containing vehicle (0.1 % DMSO). B: ACHN and Caki-1 cells were treated with vary concentrations of CPS for 24 h. Then, cells were collected and analyzed by flow cytometry analysis. C: The quantitative data showed the percentage of apoptotic cells in (C). *,# P < 0.05, **,##,$$,&&P < 0.01, ***,###,$$$,&&&P < 0.001; bars, SD. One-Way ANOVA was used for the data analysis. (JPG 800 kb) [file 12885_2016_2831_MOESM2_ESM.jpg]

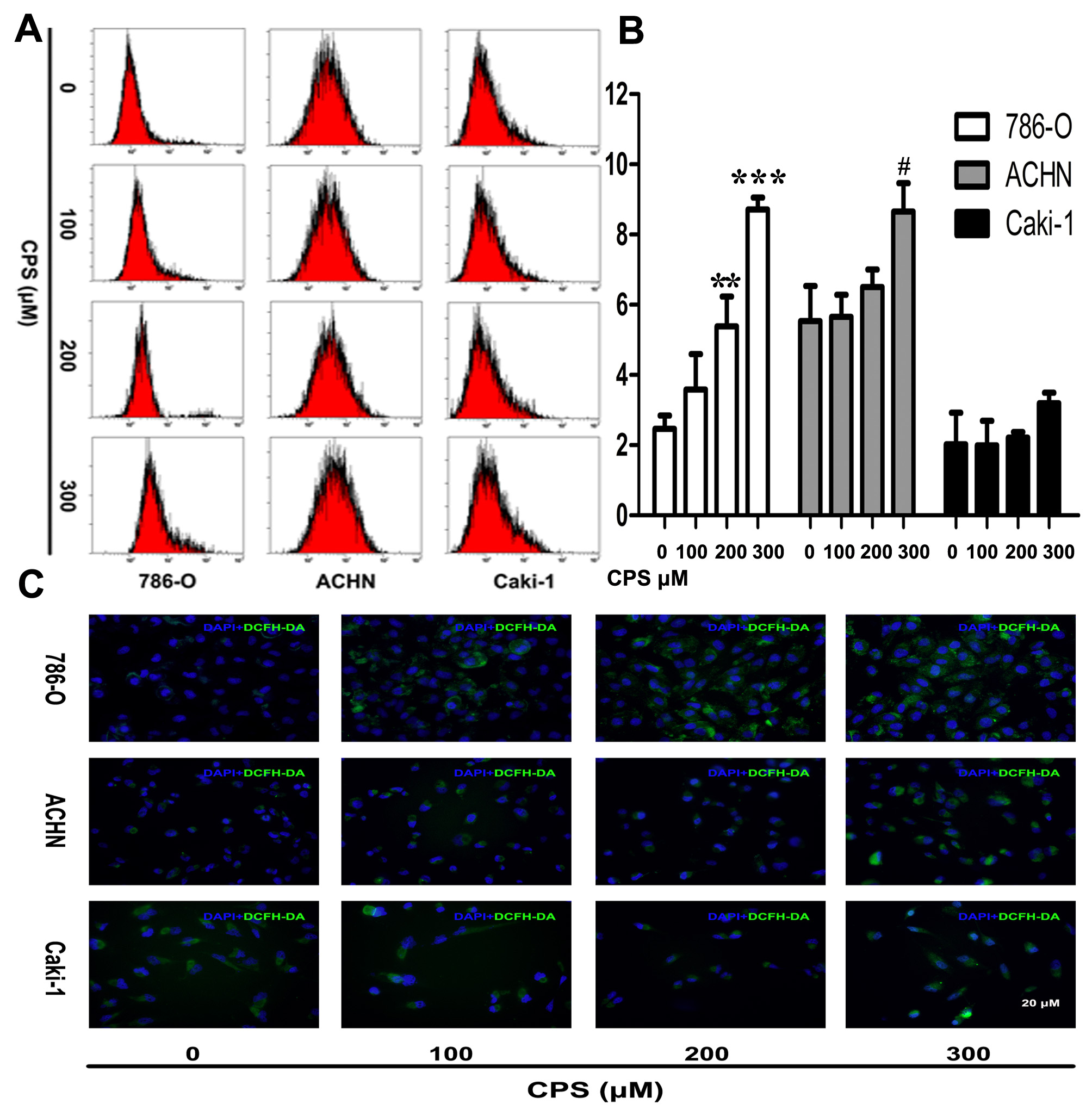

Supplement: Additional file 3: Figure S2. — CPS increased ROS Generation in RCC cells. A: Flow cytometry analysis for 786-O, ACHN, and Caki-1 cells stained with DCFH-DA, and statistically analyzed in (B), revealing an increased ROS in the RCC cells. C: DCFH-DA stained (green) 786-O, ACHN, and Caki-1 cells after CPS and vehicle treatment. Nuclears were stained by DAPI (blue). The images were photographed by fluorescence microscope and scale bars for (C) are 20 μm. # P < 0.05, *P < 0.01, ***P < 0.001; bars, SD. One-Way ANOVA was used for the data analysis. (JPG 587 kb) [file 12885_2016_2831_MOESM3_ESM.jpg]

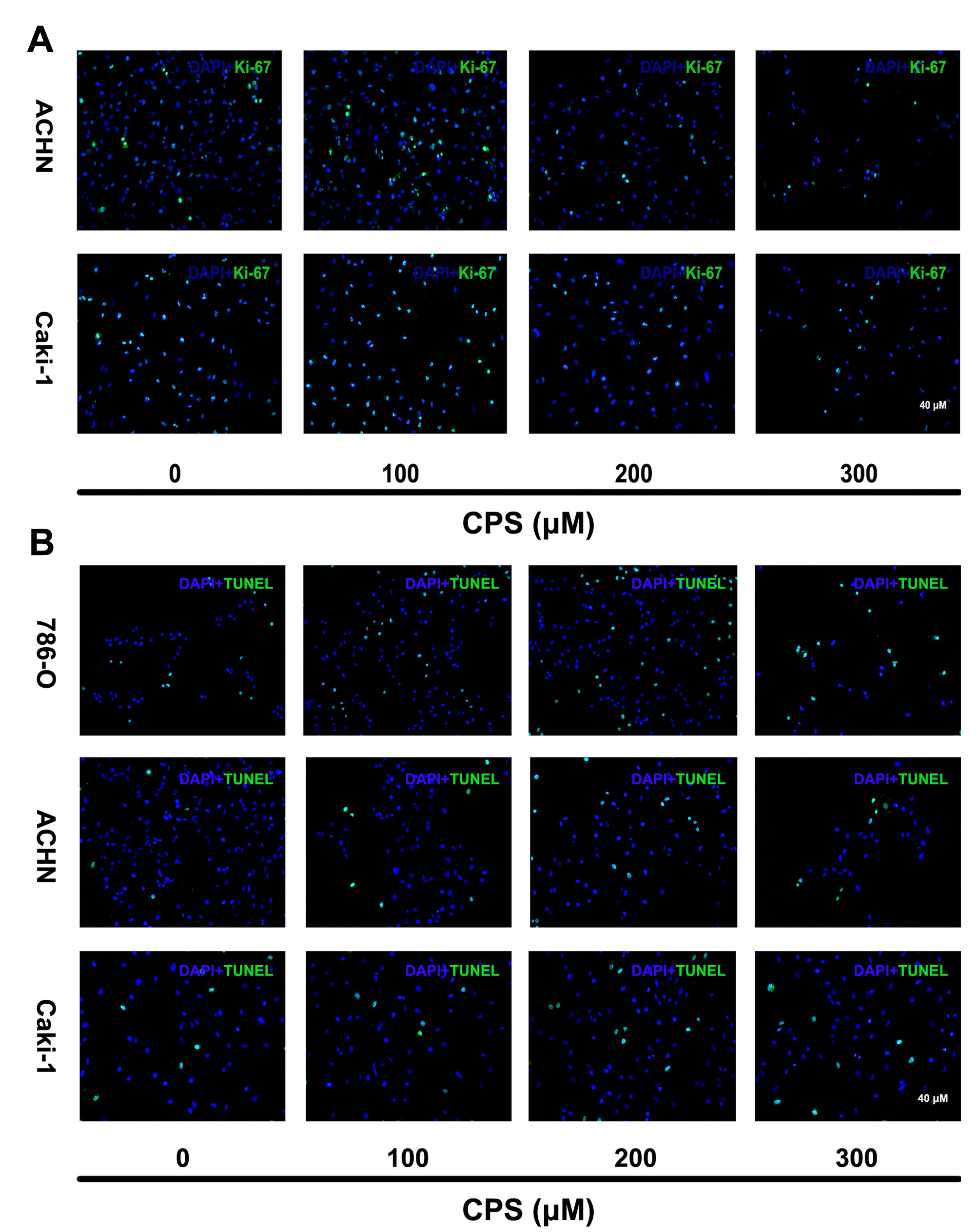

Supplement: Additional file 4: Figure S3. — CPS decreased the expression of Ki-67 but increased the TUNEL staining in RCC cells. A: Immunofluorescence staining of Ki-67 (green) in ACHN, and Caki-1 cells after CPS and vehicle treatment. B: Immunofluorescence staining of TUNEL (green) in 786-O, ACHN, and Caki-1 cells. Nuclears were stained by DAPI (blue). The images were photographed by fluorescence microscopy. The scale bar for C and D is 20 μm. (JPG 693 kb) [file 12885_2016_2831_MOESM4_ESM.jpg]
